# Diagnostic accuracy of two commercially available rapid assays for detection of IgG and IgM antibodies to SARS-CoV-2 compared to ELISA in a low-prevalence population

**DOI:** 10.3205/dgkh000363

**Published:** 2020-11-11

**Authors:** Klaus Hackner, Peter Errhalt, Martin Willheim, Felix Schragel, Maria-Anna Grasl, Jasmina Lagumdzija, Waltraud Riegler, Michael Ecker, Matthias Wechdorn, Florian Thalhammer, Ojan Assadian

**Affiliations:** 1 Department of Pneumology, University Hospital Krems, Karl Landsteiner University of Health Sciences, Krems, Austria; 2 Institute of Laboratory Medicine, University Hospital St. Poelten, Karl Landsteiner University of Health Sciences, St. Poelten, Austria; 3 Institute of Laboratory Medicine, University Hospital Krems, Karl Landsteiner University of Health Sciences, Krems, Austria; 4 Department of Medicine I, Division of Infectious Diseases and Tropical Medicine, Medical University of Vienna, Vienna, Austria; 5 Department for Infection Control and Hospital Epidemiology, Medical University of Vienna, Vienna, Austria; 6 Institute for Skin Integrity and Infection Prevention, School of Human and Health Sciences, University of Huddersfield, Huddersfield, UK

**Keywords:** COVID-19, anti-SARS-CoV2-antibodies, ELISA, point-of-care testing

## Abstract

**Background:** New commercially available point-of-care (POC) immunodiagnostic tests are appearing, which may yield rapid results for anti-SARS-CoV-2 antibodies. The aim of this study was to evaluate the diagnostic accuracy of rapid antibody detection tests compared to a validated laboratory-based enzyme-linked immunosorbent assay (ELISA) and to investigate infections amongst healthcare workers (HCWs) after unprotected close contact to COVID-19 patients.

**Methods:** Blood serum and whole blood of 130 participants were tested with NADAL^®^ COVID-19 IgG/IgM rapid test and mö-screen 2019-NCOV coronavirus test against a validated ELISA test. Infection status was evaluated using real-time polymerase-chain-reaction.

**Results:** Acute COVID-19 infection was detected in 2.4% of exposed HCWs. Antibody tests showed an overall frequency of IgG and IgM in 5.3%, with 1.6% asymptomatic infections. The NADAL^®^ test showed a sensitivity (IgM/IgG) of 100% (100%/100%), a specificity (IgM/IgG) of 98.8% (97.6%/100 %), a PPV of 76.9% (57.1%/100%), an NPV of 100% (100%/100%), and a diagnostic accuracy of 98.8% (97.7%/100%). The mö-screen test had a sensitivity (IgM/IgG) of 90.9% (80%/100%), a specificity (IgM/IgG) of 98.8% (97.6%/100%), a PPV of 76.9% (57.1%/100%), an NPV of 99.6% (99.2%/100%), and a diagnostic accuracy of 98.5% (96.9%/100%).

**Conclusions:** The frequency of COVID-19 infections in HCWs after unprotected close contact is higher than in the general population of a low-prevalence country. Both POC tests were useful for detecting IgG, but did not perform well for IgM, mainly due to false positive results.

## Zusammenfassung

**Hintergrund:** Es erscheinen neue im Handel erhältliche immundiagnostische Point-of-Care-Tests (POC), die schnelle Ergebnisse für Anti-SARS-CoV-2-Antikörper liefern können. Ziel dieser Studie war es, die diagnostische Leistungsfähigkeit von Schnelltests zum Nachweis von Antikörpern im Vergleich zu einem validierten ELISA (Enzyme-Linked Immunosorbent Assay) zu bewerten und Infektionen bei Beschäftigten im Gesundheitswesen nach ungeschütztem engen Kontakt mit COVID-19-Patienten zu detektieren.

**Methoden:** Blutserum und Vollblut von 130 Teilnehmern wurden mit dem NADAL^®^ COVID-19 IgG/IgM-Schnelltest und dem Mö-screen 2019-NCOV Coronavirus-Test gegen einen validierten ELISA-Test untersucht. Der Infektionsstatus wurde unter Verwendung einer Echtzeit-Polymerase-Kettenreaktion (RT-PCR) bewertet.

**Ergebnisse:** Eine akute COVID-19-Infektion wurde bei 2,4% der exponierten Mitarbeiterinnen festgestellt. Antikörpertests zeigten eine Gesamthäufigkeit von IgG und IgM in 5,3% bei 1,6% asymptomatischen Infektionen. Der NADAL^®^-Test zeigte eine Sensitivität (IgM/IgG) von 100% (100%/100%), eine Spezifität (IgM/IgG) von 98,8% (97,6%/100%), einen PPV von 76,9% (57,1%/100%), ein NPV von 100% (100%/100%) und eine diagnostische Genauigkeit von 98,8% (97,7%/100%). Der Mö-Screen-Test hatte eine Sensitivität (IgM/IgG) von 90,9% (80%/100%), eine Spezifität (IgM/IgG) von 98,8% (97,6%/100%), einen PPV von 76,9% (57,1%/100%), ein Kapitalwert von 99,6% (99,2%/100%) und eine diagnostische Genauigkeit von 98,5% (96,9%/100%).

**Schlussfolgerung:** Die Häufigkeit von COVID-19-Infektionen bei Mitarbeitern im Gesundheitswesen nach ungeschütztem engem Kontakt ist höher als in der Allgemeinbevölkerung eines Landes mit niedriger Prävalenz. Beide POC-Tests waren zum Nachweis von IgG nützlich, zeigten jedoch keine gute Leistungsfähigkeit für den Nachweis von IgM, hauptsächlich aufgrund falsch positiver Ergebnisse.

## Introduction

In December 2019, a novel Coronavirus, designated SARS-CoV-2, emerged in Wuhan, China, and has caused a global pandemic of human respiratory disease, now termed COVID-19 [1], [2]. The first case of an infection outside China was reported on January 13, 2020, in Thailand. On February 25, 2020, the virus has extended to Austria, with a total of 17,341 positive tested individuals until June 22^nd^ 2020.

Immediate and early implementation of mitigation strategies, including maximum reduction of social contacts, large-scale testing combined with contact tracing, and isolation of positive cases over a 14-days-quarantine period rapidly reduced the number of further hospitalizations and deaths due to COVID-19 in Austria.

So far, aside of epidemiological case-definition, molecular diagnostic techniques allowing the specific detection of viral nucleic acids were intensively used to identify positive individuals, and real-time polymerase chain reaction (RT-PCR) from specimens obtained from the upper or lower respiratory track is still considered the golden standard for establishing the diagnosis of COVID-19 [3], [4]. 

However, with the emergence of the first results of immunological studies of Chinese COVID-19 patients [5], [6], [7], [8], and the development of specific anti-SARS-CoV-2 IgM/IgG serological assays [9] it may now be possible to study immune responses more closely. Furthermore, sero-surveys may assist to determine the frequency of infection in an affected population. Additionally, new commercially available point-of-care (POC) immunodiagnostic tests are developing, which may provide rapid results for anti-SARS-CoV-2 IgG/IgM antibodies within 15 minutes [10], [11].

For COVID-19, it has been reported that the strength of human antibody response depends on a number of factors, including age, nutritional status, severity of disease, and certain medications or co-infections suppressing the immune system [7], [12], [13]. An increasing number of studies suggest that the majority of patients develop antibody response only in the second week after onset of COVID-19 specific symptoms [6], [7], [8], [12], [14], [15]. Yet, in some patients with RT-PCR verified COVID-19 infection, weak, late, or absent antibody responses have been reported [7], [12]. Antibody detection tests targeting SARS-CoV-2 may also cross-react with other pathogens, including other human coronaviruses [12], [16], [17]. Moreover, sensitivity and specificity of rapid-detection antibody kits have been only validated against PCR methods, but not against a validated ELISA test, and their true diagnostic performance is not well known in low-prevalence populations, such as currently in Austria.

Based on the current evidence, the World Health Organization (WHO) does not recommend the use of rapid antibody detecting tests for diagnosis of suspected cases, yet encourages the continuation of studies to establish their use in disease surveillance and epidemiologic research [18].

Based on the above, the aims of our study were:

1. to determine the rate of asymptomatic infections in a cohort of HCWs after unprotected and close exposure to patients with COVID-19, and
2. to determine their immune status for anti-SARS-CoV-2 antibodies using two different POC immunodiagnostic tests and a serological test assay, and finally to
3. calculate sensitivity and specificity of the POC tests, compared to a validated qualitative ELISA test.

## Methods

### Participants and study design

All participants were recruited at the University Hospital Krems, Austria, in April 2020. The participants belonged to hospital staff (e.g. medical doctors, nurses, biomedical analysts, and physiotherapists), with known close contact to COVID-19 patients without wearing personal protective equipment, mainly due to a primarily negative (but repeated and subsequently positive) RT-PCR-result and/or lack of symptoms of the patients. The date of contact was collected, and the contact was classified as either *category I* (“High-risk-exposure”, i.e. being within less than two meters for a prolonged period of time, or close contact during aerosol-generating procedures) or* category II* (“Low-risk-exposure”, i.e. contact beyond a distance of two meters for less than 15 minutes). A nasopharyngeal swab sample and blood samples (14 mL) were collected simultaneously from every participant. Five participants already fully recovered from a previous RT-PCR-confirmed COVID-19 infection, with a mean time from diagnosis to study inclusion of 39 days. Test samples were obtained in the same manner as from the collective with unknown status (n=125). Sample collection was performed with approval and in accordance with the local ethics authority of the study site (Ethic Committee of the Federal State Lower Austria, GS1-EK-3/168-2020). All participants gave their written informed consent for providing samples and data analysis.

### Real-time RT-PCR assay

Nasopharyngeal swab samples were tested for SARS-CoV-2 at the Institute for Laboratory Medicine, University Hospital St. Poelten, Austria, following WHO protocol for RT-PCR. All samples were processed immediately or stored at 2–8°C and processed within 48 hours after collection.

### ELISA

A recently developed serological enzyme-linked immunosorbent assay (ELISA) using the nucleocapsid (N) protein of the virus was applied to identify anti-SARS-CoV-2 IgG. A further assay utilizing the “IgM capture” method on microplate-based enzyme immunoassay technique was applied to detect anti-SARS-CoV-2 IgM. Both assays (supplied by Epitope Diagnostics Inc., San Diego, USA) were carried out according to the instructions of the manufacturer. For detection of anti-SARS-CoV-2 IgG, assay controls and 1:100 diluted human serum samples were added to the microtiter wells of a microplate that was coated with COVID-19 recombinant full-length N protein. After the first incubation period, the unbound protein matrix is removed with a subsequent washing step. A horseradish peroxidase (HRP)-labeled polyclonal goat anti-human IgG tracer antibody is added to each well. After an incubation period, an immunocomplex of “SARS-CoV-2 recombinant antigen – human anti-SARS-CoV-2 IgG antibody – HRP-labeled anti-human IgG tracer antibody” was formed if there was specific SARS-CoV-2 IgG antibody present in the specimen. The unbound tracer antibody was removed by the subsequent washing step. HRP-labeled tracer antibody bound to the well was then incubated with a substrate solution in a timed reaction and then measured in a spectrophotometric microplate reader. The enzymatic activity of the tracer antibody bound to the anti-SARS-CoV-2 IgG on the wall of the microtiter well was proportional to the amount of the anti-SARS-CoV-2 IgG antibody level in the tested specimen.

For detection of SARS-CoV-2 specific IgM antibodies an assay utilizing the “IgM capture” method on microplate-based enzyme immunoassay technique was applied. Assay controls and 1:10 diluted human serum samples were added to the microtiter wells of a microplate that was coated with an anti-human IgM specific antibody. After the first incubation period, the unbound protein matrix was removed with a subsequent washing step. HRP-labeled recombinant SARS-CoV-2 antigen was added to each well. After incubation, an immunocomplex of “Anti-IgM antibody – human SARS-CoV-2 IgM antibody – HRP-labeled SARS-CoV-2 antigen” was formed if there was novel SARS-CoV-2 IgM antibody present. The unbound tracer antigen was removed by the subsequent washing step. HRP-labeled SARS-CoV-2 antigen tracer bound to the well was then incubated with a substrate solution in a timed reaction and then measured in a spectrophotometric microplate reader. The enzymatic activity of the tracer antigen bound to the SARS-CoV-2 IgM on the wall of the microtiter well was proportional to the amount of the coronavirus IgM antibody level in the tested materials.

### Lateral flow chromatographic immunoassay (point-of-care antibody test)

Blood serum and whole blood was subjected to two different lateral flow chromatographic immunoassays for the qualitative detection of anti-SARS-CoV-2 IgG and IgM (NADAL^®^ COVID-19 IgG/IgM rapid test, nal von minden GmbH, Moers, Germany; and mö-screen 2019-NCOV coronavirus test, möLab GmbH, Langenfeld, Germany), according to the instructions of the manufacturers. In brief, 10 µL of serum or whole blood samples were added onto the specimen well followed by 80 µL of buffer onto the buffer well. After ten minutes of incubation, viral IgM and/or IgG containing positive samples could show up by positive test lines in addition to the control line. Results were rated as negative after an incubation time of 20 minutes precisely. Negative samples developed only a control line. According to the manufacturers, in the early stages of infection (three to seven days) anti-SARS-CoV-2 IgG and IgM may be below the detection limit of the test. 

### Statistical analysis

All statistical data were analyzed with IBM SPSS Statistics software, version 23 (SPSS, IBM Corporation, Armonk, NY). Specificity, sensitivity, positive predictive value, negative predictive value, and accuracy were calculated via contingency table. Confidence intervals for sensitivity, specificity, and accuracy were “exact” Clopper-Pearson confidence intervals. Confidence intervals for the predictive values are the standard logit confidence intervals given by Mercaldo et al [19]. The McNemar test was used to evaluate difference of serum and whole blood. Differences were considered statistically significant if *p* was <0.05. 

## Results

130 participants were included into the study, with 76.9% females (n=100) and 23.1% males (n=30). The median age was 41 years (ranging from 19 to 64). A majority of the participants (65.4%) were nurses, 23.8% were medical doctors, and 10.8% were employed in other fields (e.g. biomedical analysts, physiotherapists, etc.). 101 participants (77.7%) had a high-risk-exposition (*category I*), while 29 participants (22.3%) were classified as low-risk-exposed (*category II*). The mean number of days from the contact to a COVID-19 patient without personal protective equipment until sampling was 19.4 (±13.9) days (Table 1 (Tab. 1)).

Table 2 (Tab. 2) summarizes the results of the RT-PCR, ELISA, and the POC tests, and Table 3 (Tab. 3) gives details on all participants with their respective results. Four participants were positively tested with RT-PCR (3.1% of the overall study group), one with a history of confirmed and recovered COVID-19 infection, but three with a previous unknown status and therefore newly detected infection (2.4% of the unknown status collective). These three participants failed to show IgG or IgM antibodies by any test, but later developed clinical symptoms. 

Test concordance (IgM+IgG) of the NADAL^®^ COVID-19 IgG/IgM rapid test for serum vs. whole blood was 96.9% and for the mö-screen 2019-NCOV coronavirus test 97.7%. Comparison of the results of the two POC tests showed 99.2% concordance for serum and 100% for whole blood. Test concordance of the both POC tests compared to the reference standard ELISA was slightly higher when using serum instead of whole blood (NADAL^®^ COVID-19 IgG/IgM rapid test 97.7% vs. 96.2%; mö-screen 2019-NCOV coronavirus test 96.9% vs. 96.2%), however the differences were not statistically significant.

Using ELISA, five participants with a history of a COVID-19 infection (two with IgM+IgG, two with IgG only, one with IgM only), and two participants of unknown infection status were positively tested for anti-SARS-CoV-2 antibodies (one with IgM+IgG, one with IgG only). Both did not experience any clinical symptoms and therefore were defined as asymptomatically infected (=1.6% of the participants with unknown COVID-19 status, and 1.9% of the participants with a *categoryI*high-risk exposure contact). 

Table 4 (Tab. 4) and Table 5 (Tab. 5) depict the performance of the two POC tests compared to ELISA, when using blood serum. The NADAL^®^ COVID-19 IgG/IgM rapid test showed a sensitivity (IgM/IgG) of 100% (100%/100%), a specificity (IgM/ IgG) of 98.8% (97.6%/100%), a PPV of 76.9% (57.1%/100%), an NPV of 100% (100%/100%), and a diagnostic accuracy of 98.8% (97.7%/100%). The mö-screen 2019-NCOV coronavirus test had a sensitivity (IgM/IgG) of 90.9% (80%/100%), a specificity (IgM/IgG) of 98.8% (97.6%/100%), a PPV of 76.9% (57.1%/100%), an NPV of 99.6% (99.2%/100%), and a diagnostic accuracy of 98.5% (96.9%/100%).

## Discussion

To our knowledge, this is the first study analyzing the number of COVID-19 infections and development of antibodies against SARS-CoV-2 among HCWs after high-risk-exposition to COVID-19 patients, without wearing personal protective equipment. Additionally, the diagnostic performance of two commercially available POC lateral flow chromatographic immunoassay tests compared to a reference standard ELISA test was investigated.

The prevalence of newly detected (acute) COVID-19 infections among participants with unknown infection status was 2.4% (n=3). These three participants soon developed symptoms, with a mean time of their *category I* exposition to testing of 4.5 days. Furthermore, these staff members were all employed at the same department, and contact tracking suggests that they were all infected by the same COVID-19 patient. Another attending nurse of this department was negatively tested during the study participation, but developed symptoms after 3 days, which led to another RT-PCR investigation, with a positive result.

In early April 2020, a study initiated by the Austrian government was launched to estimate the prevalence of acute infections with COVID-19 among non-hospitalized inhabitants in Austria with 1,544 random samples. This study found a prevalence of 0.33% (95% CI 0.12–0.76) positively tested individuals [20]. Compared to this result, the current study showed a higher proportion of positively tested participants (0.33% in the general population vs. 3.9 % in this high-risk-exposition group with *category I* contact). We were able to detect two participants with anti-SARS-CoV-2 antibodies with an asymptomatic infection (mean number of 47 days from contact to testing) in the collective of unknown infection status (1.6%). This finding is even lower compared to recent results of an Austrian anti-SARS-CoV-2 antibody study performed in 27 high-prevalence COVID-19 municipalities on 269 random participants, showing antibodies in 4.71% of the sample (95% CI 1.36–7.97) [21]. Therefore, it may be assumed that the overall rate of immunization in a general and a high-risk-exposition population is low, and herd immunity is far outside reach.

We did not find a difference among the two investigated POC tests when using whole blood. However, optical assessment of the result appeared slightly superior with mö-screen 2019-NCOV coronavirus test. When using blood serum instead of whole blood, both POC tests showed high concordance compared to ELISA, yet with no statistically significant difference. Still, although centrifugation is an additional time-demanding work step, we suggest preferring serum over whole blood whenever possible.

Both POC tests were surprisingly accurate in detecting IgG, when compared to ELISA (PPV and NPV, both 1.0). However, for IgM antibodies, both tests showed also false positive or false negative results, ensuing a PPV of 0.57. However, despite the limited sample size, these performance results indicate that POC tests might be a useful tool to evaluate a previous COVID-19 infection, especially when 3, better 4 weeks have passed after onset of symptoms and/or a potential contact and testing. As reported, a SARS-CoV-2-infected person may start to replicate the virus on day 4 to 5, and a nasopharyngeal swab sample collection has a high probability for a positive result [6], [7], [8]. Such a person is infective (starting two to three days prior to symptoms [22]) and may spread the virus. The presence of the virus may be verified with RT-PCR for 14 days (up to 40 days), but approximately two weeks after exposition, the first SARS-CoV-2 IgM peak is reached. By then, the number of long-lasting SARS-CoV-2 IgG is increasing, with a possible climax five to six weeks after the infection or exposition, respectively. In any ways, the antibody kinetics in COVID-19 is unclear, and there is a need for further kinetics studies among different groups at several timepoints.

This study has potential limitations. First, the mean range from time of contact to testing were 18 days, ranging from 3 to 47 days. Especially for participants with a short interval, we cannot exclude that the timeframe was too short for antibody development. Another examination in three to five weeks would be a useful way to rule this out, or to describe the longitudinal course of the antibody development. Three participants also showed borderline ELISA results for anti-SARS-CoV-2 antibodies (generally counted as ‘negative’). Consecutive testing could determine the subsequent immunization status of this participants. Second, this is a monocentric study with limited sample size, and larger scaled studies would be useful to confirm our results.

In conclusion, this study showed a higher rate of acute COVID-19 infections in a high-risk-exposition group compared to the general low-prevalence Austrian population. However, detection rates of anti-SARS-CoV-2 IgG and IgM were low, suggesting an overall low number of asymptomatic infections, even among health-care workers with known high-risk-exposition to the virus. Both POC tests proved to be very useful for detecting IgG, but did not perform as well for detecting IgM, mainly due to a proportion of false positive results. The use of blood serum might be superior to the use of whole blood with POC tests and thus should be preferred.

## Notes

### Competing interests

The authors declare that they have no competing interests.

### Ethical statement

Sample collection was performed with approval and in accordance with the local ethics authority of the study site (Ethic Committee of the Federal State Lower Austria, GS1-EK-3/168-2020). All participants gave their written informed consent for providing samples and data analysis.

Trial registration number: DRKS00022083

### Funding

This research did not receive any specific grant from funding agencies in the public, commercial, or not-for-profit sectors. Consumable costs were covered through the routine diagnostic and therapeutic budget of the Lower Austrian State Health Agency.

## Figures and Tables

**Table 1:**
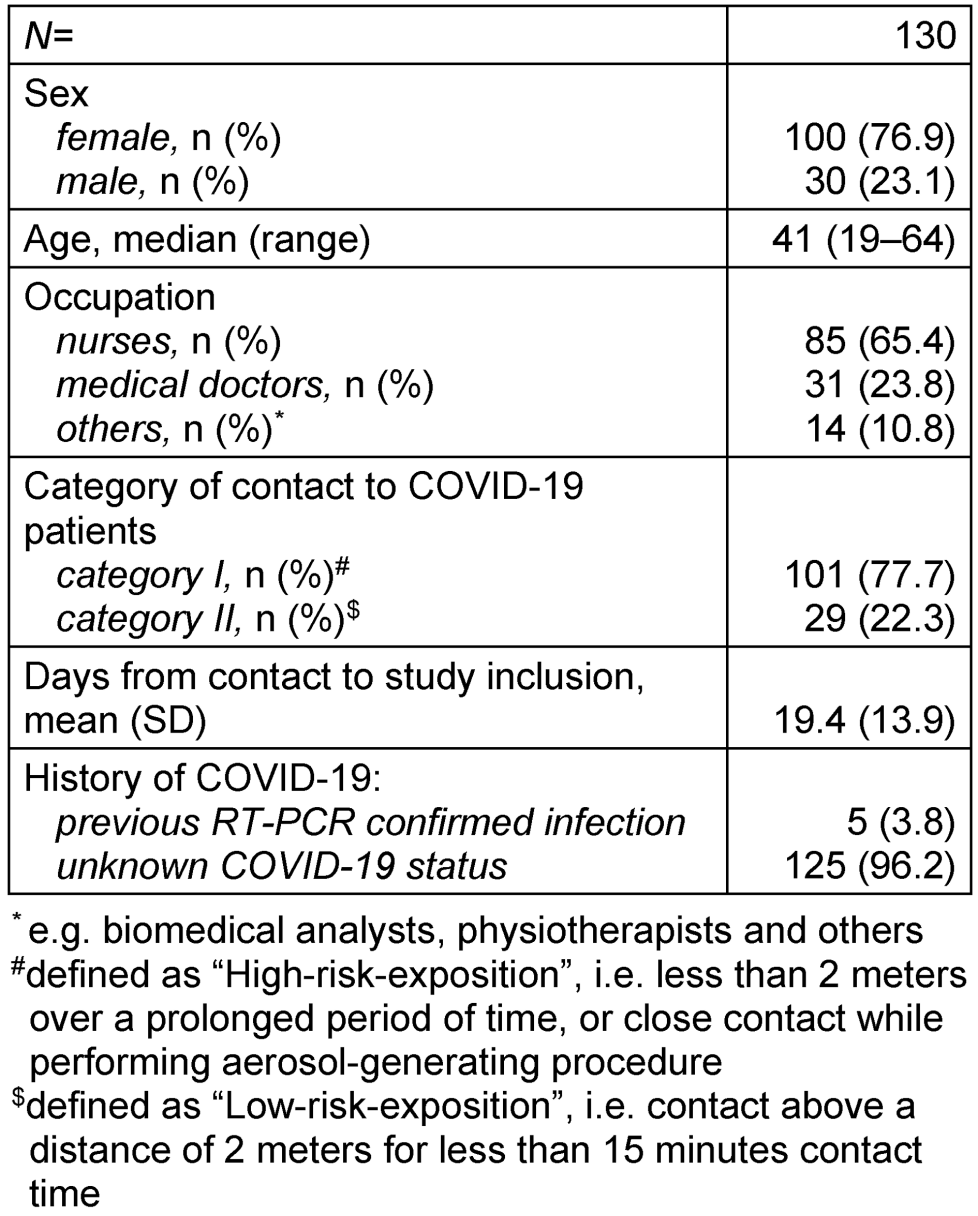
Baseline characteristics

**Table 2:**
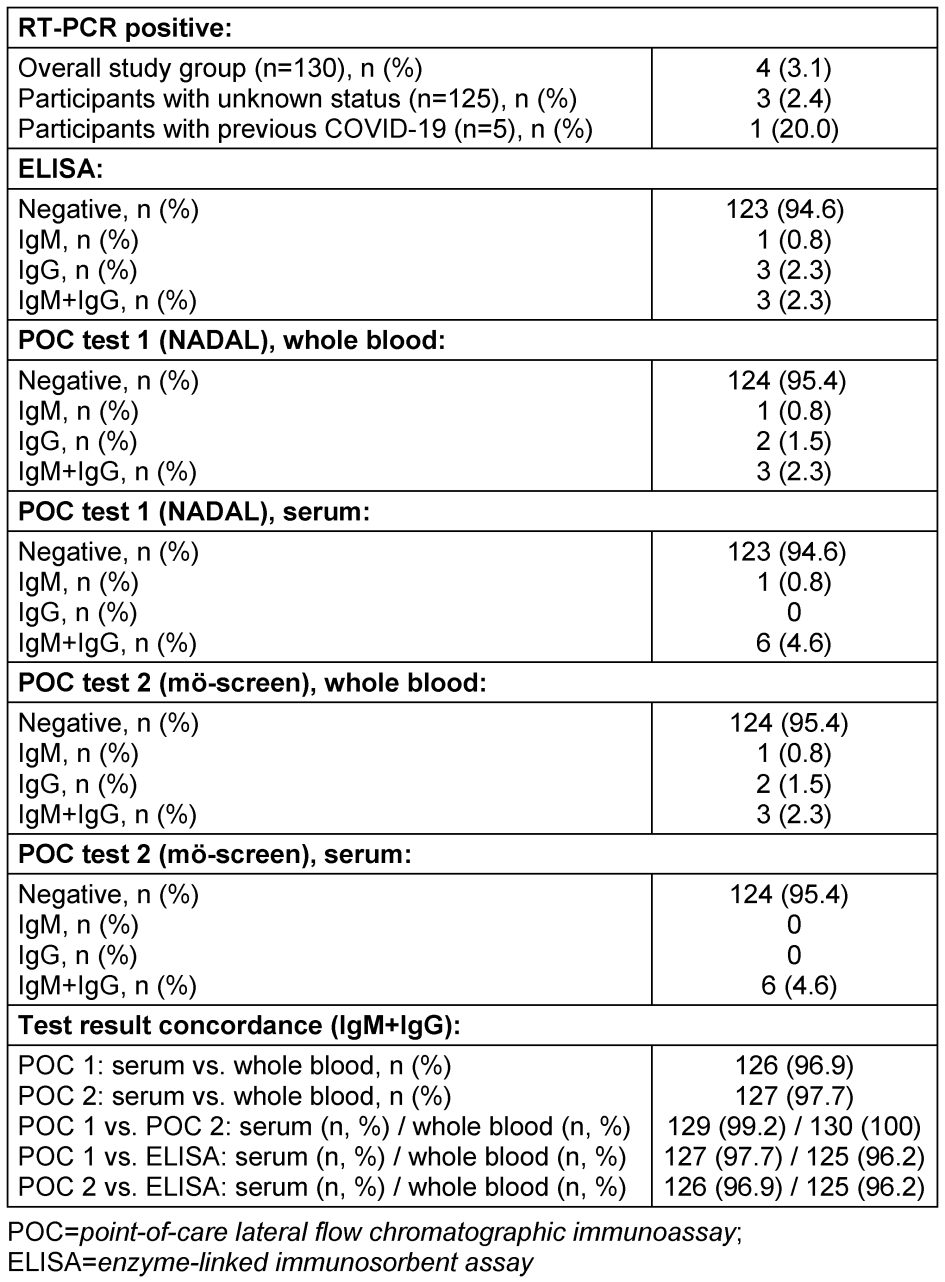
Overview of the main results of RT-PCR, ELISA and both point-of-care lateral flow chromatographic immunoassay, and test concordance among different assays and specimens (blood serum vs. whole blood)

**Table 3:**
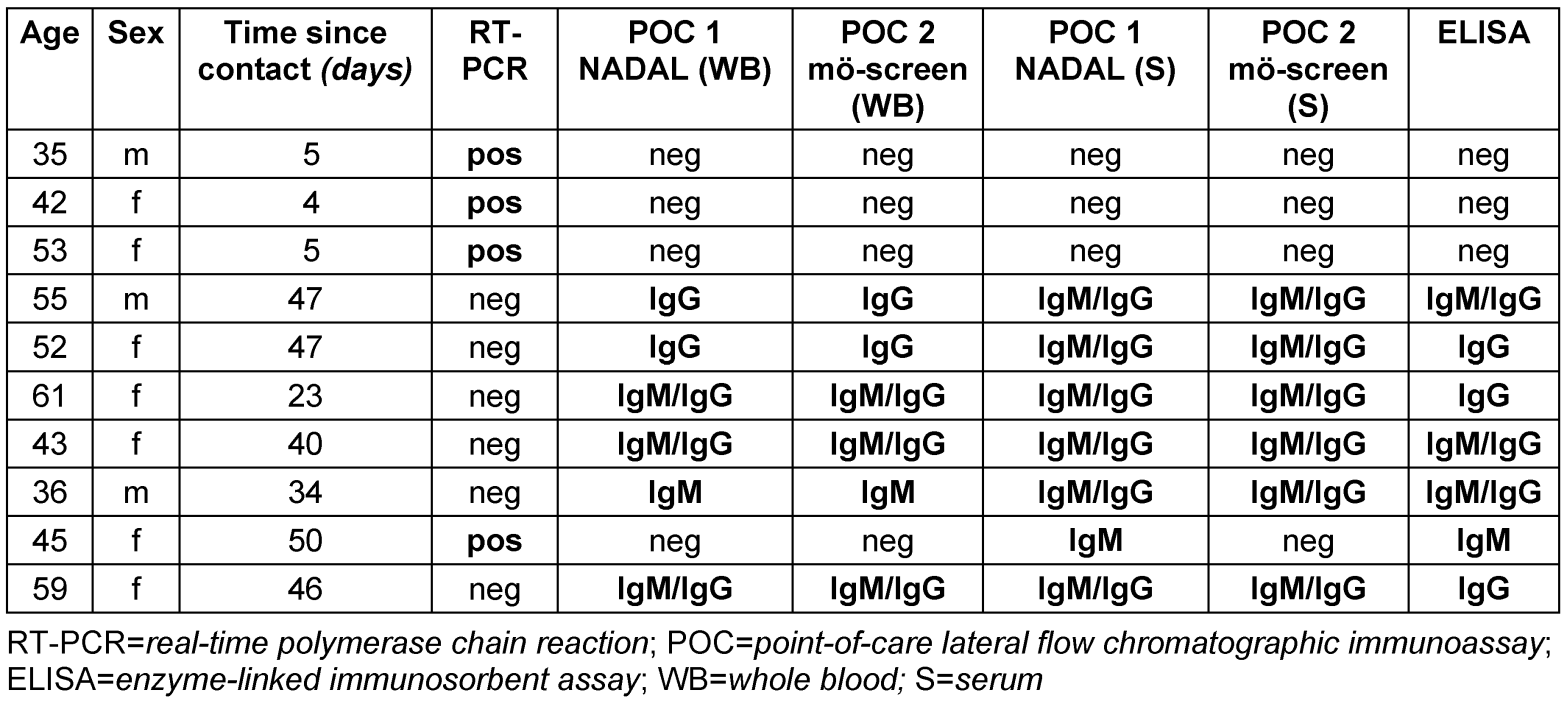
Details of study participants with any positive result for SARS-CoV-2 infection and/or anti-SARS-CoV-2 antibodies

**Table 4:**
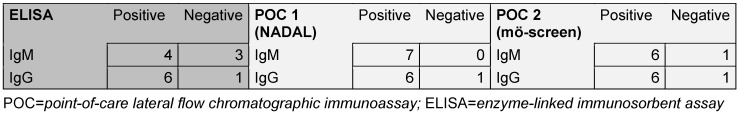
Table 4. Comparison of lateral flow chromatographic immunoassay (point-of-care antibody tests: NADAL^®^ COVID-19 IgG/IgM rapid test; mö-screen 2019-NCOV coronavirus test) and reference standard for anti-SARS-CoV-2 antibodies (ELISA), using blood serum, in seven participants with detected SARS-CoV-2 antibodies

**Table 5:**
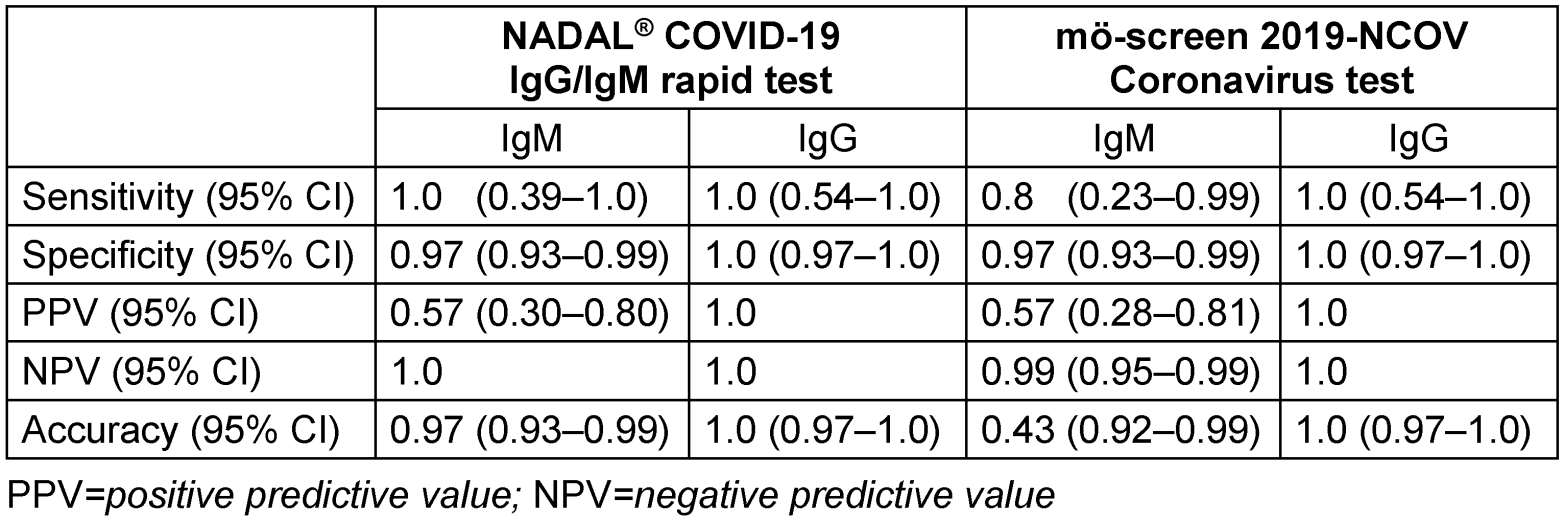
Result overview: Diagnostic accuracy of lateral flow chromatographic immunoassay (point-of-care antibody tests: NADAL^®^ COVID-19 IgG/IgM rapid test; mö-screen 2019-NCOV coronavirus test) vs. reference standard for anti-SARS-CoV-2 antibodies (ELISA), using blood serum
